# Mitochondrial transcript maturation and its disorders

**DOI:** 10.1007/s10545-015-9859-z

**Published:** 2015-05-28

**Authors:** Lindsey Van Haute, Sarah F. Pearce, Christopher A. Powell, Aaron R. D’Souza, Thomas J. Nicholls, Michal Minczuk

**Affiliations:** MRC Mitochondrial Biology Unit, Hills Road, Cambridge, CB2 0XY UK; Department of Medical Biochemistry and Cell Biology, University of Gothenburg, Gothenburg, Sweden

## Abstract

Mitochondrial respiratory chain deficiencies exhibit a wide spectrum of clinical presentations owing to defective mitochondrial energy production through oxidative phosphorylation. These defects can be caused by either mutations in the mitochondrial DNA (mtDNA) or mutations in nuclear genes coding for mitochondrially-targeted proteins. The underlying pathomechanisms can affect numerous pathways involved in mitochondrial biology including expression of mtDNA-encoded genes. Expression of the mitochondrial genes is extensively regulated at the post-transcriptional stage and entails nucleolytic cleavage of precursor RNAs, RNA nucleotide modifications, RNA polyadenylation, RNA quality and stability control. These processes ensure proper mitochondrial RNA (mtRNA) function, and are regulated by dedicated, nuclear-encoded enzymes. Recent growing evidence suggests that mutations in these nuclear genes, leading to incorrect maturation of RNAs, are a cause of human mitochondrial disease. Additionally, mutations in mtDNA-encoded genes may also affect RNA maturation and are frequently associated with human disease. We review the current knowledge on a subset of nuclear-encoded genes coding for proteins involved in mitochondrial RNA maturation, for which genetic variants impacting upon mitochondrial pathophysiology have been reported. Also, primary pathological mtDNA mutations with recognised effects upon RNA processing are described.

## Introduction

**Fig. 1:**
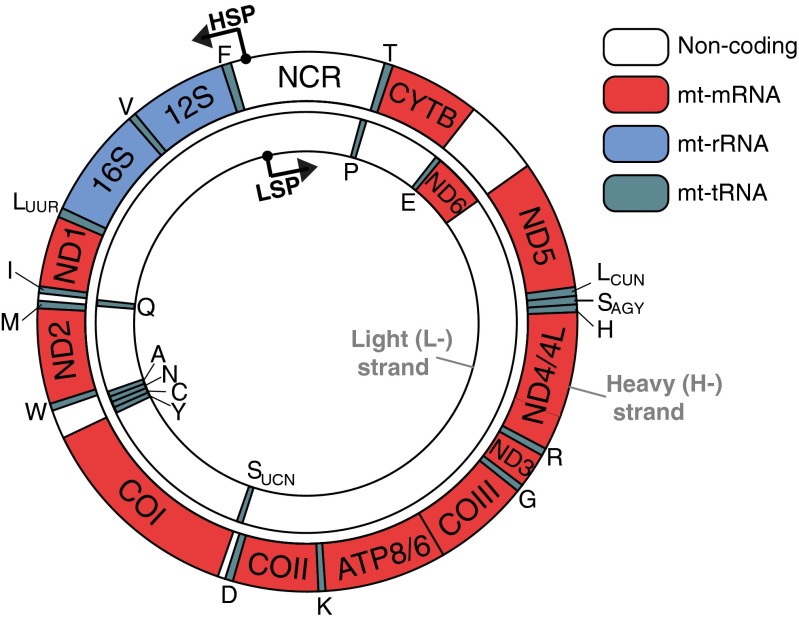
Genetic map of the mitochondrial genome. The organisation of the genes on human mitochondrial genome is shown. The two strands of the human mtDNA, denoted light (L-) and heavy (H-), code for 2 mt-rRNAs (*blue*), 22 mt-tRNAs (*green*) and 11 mt-mRNA molecules (*red*). The 11 mt-mRNAs encode 13 polypeptides of the electron transport chain and ATP synthase, where open reading frames of ATP8/ATP6 and ND4/ND4L overlap and are contained within bicistronic mRNAs. The main non-coding region (NCR) contains promoters for transcription of the H-strand and L-strand, HSP and LSP, respectively

Mitochondria are cellular compartments present in every cell of the human body (except red blood cells) and are responsible for generating almost all of the energy needed to sustain life and to grow. In mitochondria, energy is produced in the process of oxidative phosphorylation (OXPHOS). The biogenesis of the OXPHOS system entails assembly of approximately 90 proteins into five complexes. Most of these proteins are encoded by DNA that is contained within the cell nucleus (nDNA). However, 13 of these are encoded within a small, circular genome inside mitochondria (Fig. 1). Recent research has identified defects in mitochondrial DNA (mtDNA) expression that are associated with a diverse group of human disorders characterised by impaired mitochondrial respiration (Nicholls et al 2013; Boczonadi and Horvath 2014). Many regulatory factors and pathways are involved in mitochondrial gene expression and establishing the molecular details of how defects in these processes contribute to human disease constitutes a major challenge.

In addition to messenger RNAs (mt-mRNAs), the human mitochondrial transcriptome also consists of two ribosomal RNAs (mt-rRNAs, 12S and 16S) that are a part of the mitochondrial ribosome and a complete set of 22 transfer RNAs (mt-tRNAs) (Rorbach and Minczuk 2012) (Fig. 1). All of the mitochondrial protein factors necessary for RNA synthesis, endonucleolytic processing, post-transcriptional modifications, aminoacylation, RNA stability regulation, and RNA turnover along with the factors necessary for biogenesis of the mitochondrial ribosome and translation are encoded by nDNA and imported to mitochondria upon translation in the cytosol. Based on the literature, evidence from studies of human mitochondrial proteomes (Pagliarini et al 2008; Smith et al 2012) and our unpublished predictions, we estimate that 250–300 nuclear-encoded proteins are dedicated to serve mitochondrial gene expression.

Mutations associated with mitochondrial disease are located either within nDNA or mtDNA. Most mitochondrial diseases are early onset, multi-systemic (affecting the brain, heart, skeletal muscle, liver and other organs) and often fatal. Inherited pathological mutations in genes encoding components involved in mitochondrial gene expression are generally associated with combined OXPHOS deficiencies affecting multiple enzymes involved in cellular respiration. However, isolated OXPHOS deficiencies, that affect only a specific biochemical activity of the OXPHOS system, have also been described for mutants in mitochondrial gene expression (e.g. Haack et al 2013). Many pathological mutations in the mtDNA-encoded tRNAs and rRNAs have been identified thus far (Yarham et al 2010), and given the size of mtDNA, detection of yet uncharacterised mutations is relatively straightforward. In contrast, the molecular diagnosis of patients with defects of mitochondrial gene expression due to mutations in nDNA is particularly difficult, mostly because of the high number of nuclear genes involved in these processes with only a fraction of them being characterised to date (Nicholls et al 2013; Boczonadi and Horvath 2014).

This review article describes how defects in mitochondrial RNA maturation are linked to human mitochondrial disease. We provide an overview of the mechanisms of mitochondrial transcript synthesis, endonucleolytic processing and post-transcriptional modifications of each class of mtRNA. This is followed by a description of a subset of nuclear-encoded factors involved in mtRNA metabolism, for which genetic variants impacting upon mitochondrial pathophysiology have been reported. Finally, we discuss primary pathological mtDNA mutations with recognised effects upon mt-tRNA nucleolytic processing and post-transcriptional maturation.

## Mechanisms of mitochondrial RNA maturation

**Fig. 2:**
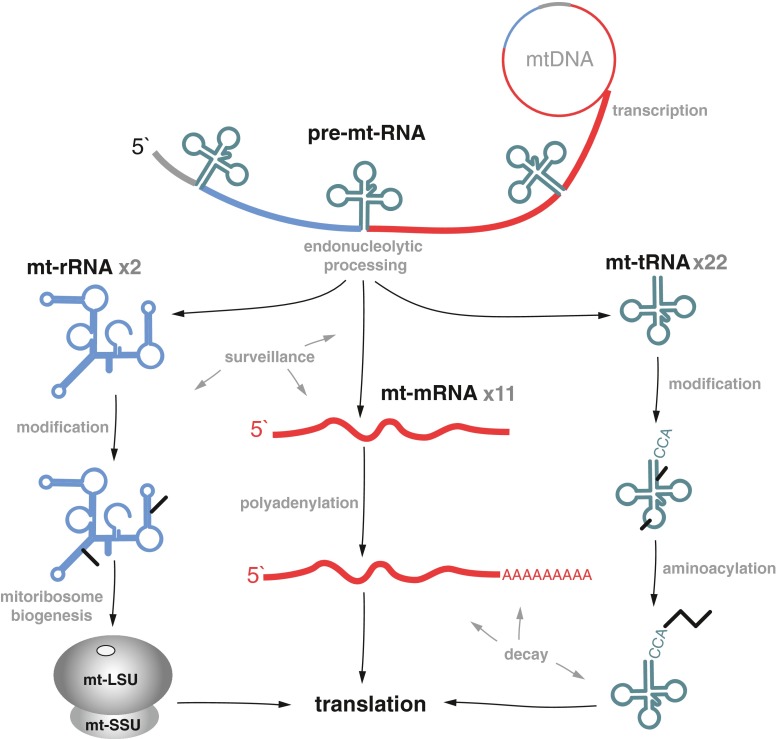
Mitochondrial RNA metabolism. The mitochondrial rRNAs (*blue*), mRNAs (*red*) and tRNAs (*green*) are transcribed from the L- and H-strands as polycistronic units that undergo endonucleolytic processing. Following the liberation of the individual mt-mRNA, mt-rRNA and mt-tRNA transcripts, they undergo post-transcriptional modifications. Several nucleotides of mt-rRNAs are modified to facilitate mitoribosome biogenesis and function. A poly(A) tail is added to mt-mRNAs, with the exception of the L-strand-encoded ND6. Mt-tRNAs undergo extensive post-transcriptional nucleotide modification, in addition to a CCA trinucleotide synthesis at the 3′ end, before being aminoacylated with a cognate amino acid. Decay and surveillance pathways have also been described for mammalian mtRNA

The generation of functional RNA molecules used for protein synthesis requires transcription, nucleolytic processing, post-transcriptional nucleotide modifications, polyadenylation of mt-mRNA and mt-tRNA-aminoacylation (Fig. 2). These steps are coordinated with RNA quality control, RNA stability regulation, biogenesis of the mitochondrial ribosome and translation activation (Fig. 2). However, the mechanistic details of how these processes occur are still unknown. In the first part of the review, before discussing human diseases associated with defects of mitochondrial RNA metabolism, we summarise the current available data on mtRNA metabolism.

### Generation of primary transcript and its endonucleolytic processing

#### Mitochondrial transcription

**Fig. 3:**
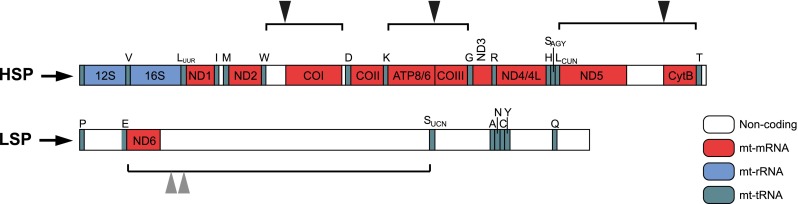
Polycistronic transcription units in mitochondria. Polycistronic precursor mitochondrial transcripts are shown. The transcript from LSP contains only the coding sequences for the ND6 subunit of complex I and eight mt-tRNAs. All other coding sequences are produced by transcription from HSP. With some exceptions (triangles), mt-rRNAs (*blue*) and mt-mRNAs (*red*) are punctuated with mt-tRNAs (*green*). The endonucleolytic processing of mt-tRNA liberates most of the mt-mRNAs and the two mt-rRNAs. The enzymatic machinery responsible for the processing at the non-canonical sites, not punctuated with mt-tRNAs, is not well investigated. ND6 mRNA shows multiple 3′ ends: either 500 nt (Slomovic et al 2005) or 30 nt (Mercer et al 2011) downstream of the translation termination codon

Transcription of the human mitochondrial genome occurs on both DNA strands, is polycistronic, and produces long transcripts of mt-mRNAs and mt-rRNAs, usually interspersed with mt-tRNAs (Ojala et al 1981). Transcription of the light strand (L-strand, Fig. 1) template occurs from a single promoter (L-strand promoter, LSP) and this long transcript encodes the ND6 gene and eight mt-tRNAs (Figs. 1 and 3) (Montoya et al 1982, 1983). The majority of initiation events from LSP are believed to terminate some 200 bases downstream from the promoter, at “conserved sequence block 2” (CSB2) (Wanrooij et al 2010). This short RNA species is used to prime replication of the heavy DNA strand (H-strand, Fig. 1) as well as the synthesis of the D-loop, whose biogenesis and function is discussed in our recent review (Nicholls and Minczuk 2014). Classically, transcription of the H-strand template is initiated from two sites (H-strand promoter 1 and 2, HSP1 and HSP2), located approximately 100 bases apart in the non-coding region (NCR) (Montoya et al 1982, 1983; Chang and Clayton 1984). HSP1 is located just upstream of the mt-tRNA^Phe^ gene, and initiation from this site produces a short transcript consisting of the two ribosomal RNAs (12S and 16S) and mt-tRNA^Val^, terminating at the mt-tRNA^Leu(UUR)^ gene. Transcription from HSP2, immediately upstream from the 12S mt-rRNA gene, bypasses this termination and produces an almost genome-length transcript of 2 mt-rRNAs, 12 mt-mRNAs and 14 mt-tRNAs. The mitochondrial transcription termination factor mTERF1 binds strongly to a tridecamer DNA sequence and has been proposed to terminate H-strand transcription at the mt-tRNA^Leu(UUR)^ gene (Christianson and Clayton 1988; Kruse et al 1989; Fernandez-Silva et al 1997). However, this was brought into doubt by the discovery that a mouse knockout of mTERF1 had normal H-strand transcription (Terzioglu et al 2013). It has been suggested that mTERF1 may actually serve to terminate the L-strand transcript, preventing the formation of antisense mt-rRNA species. The existence of HSP2 as a functional promoter in vivo is also questionable as transcription from this site is not readily detectable using established in vitro transcription systems (Litonin et al 2010). It may be the case, therefore, that only LSP and HSP1 (indicated as HSP on Fig. 1) are responsible for the transcription of all mitochondrially-encoded transcripts.

The basal protein machinery necessary for mitochondrial transcription has been well studied in humans, where RNA synthesis is performed by a single-subunit mitochondrial RNA polymerase, POLRMT, that shares homology with the RNA polymerases of the T-odd phages T3 and T7 (Masters et al 1987). POLRMT was identified through its homology with its yeast counterpart (Tiranti et al 1997). Later two homologues of the yeast mitochondrial transcription factor, mtTFB, were identified and named TFB1M and TFB2M (Falkenberg et al 2002; Cotney and Shadel 2006). Both contain an rRNA methyltransferase-like fold (Shutt and Gray 2006). TFB2M has been found to be necessary for transcription, and so appears to be the functional homologue of yeast mtTFB (Falkenberg et al 2002; Litonin et al 2010). TFB1M, on the other hand, appears to have mainly retained its dimethyltransferase function (see below) (Seidel-Rogol et al 2003; Metodiev et al 2009). Transcription in human mitochondria also requires mitochondrial transcription factor A (TFAM) (Falkenberg et al 2002; Shi et al 2012). The ability of TFAM to activate transcription is dependent upon a C-terminal tail, which is absent from its yeast homologue, Abf2p (Dairaghi et al 1995; Garstka et al 2003). Consistent with this, Abf2p is not required for yeast mitochondrial transcription, and is only involved in DNA packaging (Diffley and Stillman 1991; Parisi et al 1993).

In addition to these core factors, other proteins implicated in mitochondrial transcription include the transcription elongation factor TEFM and the large mitoribosomal subunit protein MRPL12. Acting in complex with POLRMT, TEFM is necessary for processivity of the polymerase and for increasing transcription rates by preventing a termination event at CSB2 (Minczuk et al 2011; Agaronyan et al 2015; Posse et al 2015). MRPL12 has also been found to bind POLRMT, and stimulate transcription in in vitro reactions (Wang et al 2007). This stimulatory effect was not observed in a later study from another group (Litonin et al 2010).

#### Nucleolytic processing of mitochondrial precursor RNA

Transcription of mtDNA results in polycistronic RNA molecules (Fig. 3). Most of the mt-mRNA or mt-rRNA-coding regions are separated by mt-tRNAs, which upon excision generate RNA products for translation or assembly into ribosomes (Anderson et al 1981; Ojala et al 1981). This processing mechanism is referred to as the ‘tRNA punctuation model’. RNase P and RNase Z (elaC homolog 2 (ELAC2)) are the two main factors involved in mt-tRNA excision.

The RNase P enzyme is responsible for the 5′ end endonucleolytic cleavage of mt-tRNA precursors. Unlike the previously characterised RNase P enzymes, the mitochondrial counterpart contains no catalytic RNA component. Instead, it is composed of three multifunctional mitochondrial RNase P proteins, MRPP1 (TMRT10C or RG9MTD1), MRPP2 (HSD17B10, SDR5C1) and MRPP3 (Holzmann et al 2008). MRPP1 is a m^1^G9-methylase, whereas MRPP2 is a dehydrogenase also involved in isoleucine metabolism and other cellular functions (Yang et al 2005). MRPP1 and MRPP2 form a subcomplex that also participates in mt-tRNA modification (Vilardo et al 2012) (see below). MRPP3 is responsible for hydrolysis of the RNA phosphodiester bond (Rossmanith and Holzmann 2009). Knockdown of the different protein components of the mitochondrial RNase P all cause an accumulation of precursor RNA molecules (Holzmann et al 2008). Also, MRPP1 or MRPP3 knockdown reduced steady state levels of mature mt-tRNAs and some mt-mRNAs, which had a detrimental effect on mitochondrial translation (Sanchez et al 2011).

RNase Z, encoded by *ELAC2*, is an endonuclease that completes the excision of mt-tRNAs from the primary transcript at the 3′ end. Human ELAC2 has been identified by homology to the bacterial counterpart elaC. There are two human genes encoding orthologs of elaC: ELAC1 and ELAC2. *ELAC1* encodes a short form of RNase Z, localised in the cytosol (Rossmanith 2011), whereas *ELAC2* encodes a long form of RNase Z and is restricted to eukaryotes. Alternative translation initiation of ELAC2 mRNA generates two products, of which only the longer form localises to mitochondria. In vitro, ELAC2 shows catalytic activity on both nuclear and mitochondrial pre-tRNAs (Rossmanith 2011). Knockdown of the protein causes the accumulation of mt-tRNA precursors (Brzezniak et al 2011; Sanchez et al 2011).

Other proteins have been recently implicated in precursor RNA processing in mitochondria. G-rich sequence factor 1, GRSF1, is an RNA-binding protein that co-localises with newly synthesised transcripts in mitochondrial foci named “RNA granules” (Antonicka et al 2013; Jourdain et al 2013). A decrease in some mature mitochondrial transcripts upon GRSF1 knockdown and co-localisation of GRSF1 with MRPP1 in RNA granules suggested a possible cooperative role in precursor RNA processing (Jourdain et al 2013). The discovery of RNA granules has catalysed further research on these structures, indicating very recently that they are central for post-transcriptional RNA maturation and the biogenesis of mitochondrial ribosomes (see below) (Antonicka and Shoubridge 2015; Jourdain et al 2015).

Pentatricopeptide-repeat containing (PPR) domain family of proteins member 1, PTCD1 (Small and Peeters 2000), is predicted to have a role in mtRNA metabolism. It has been suggested that this protein plays a role in coordinating 3′ processing of mt-tRNAs and that there is a direct interaction between PTCD1 and ELAC2 (Sanchez et al 2011). Additional studies have suggested a role for PTCD1 in controlling the levels of both leucine mt-tRNAs (Rackham et al 2009), which might have a role in adaptation of mitochondria to amino acid starvation (Schild et al 2014).

There are various sites in mitochondrial polycistronic transcripts that are not consistent with the ‘tRNA punctuation model’ and are cleaved independently of the presence of mt-tRNAs (Fig. 3). These include: (i) the bicistronic ATP8/6-COIII, (ii) the 5′ end of COI, (iii) the 5′ end of CytB, and (iv) the 3′ end of ND6 (Anderson et al 1981; Temperley et al 2010). There are no obvious sequence motifs within the proximal regions of COI, COIII or CytB that could serve as recognition sites for cleavage. Knockdown of ELAC2 and the components of RNase P show no effect on the levels of these non-conventional cleavage sites (Brzezniak et al 2011). PTCD2 has been implicated in the cleavage of the junction between ND5 and CytB (Fig. 3) as a PTCD2 knockout mouse accumulates the ND5-CytB precursor and do not properly assemble complex III (where CytB is the catalytic core component) (Xu et al 2008a). More recently, an RNA granule protein, FASTKD5 (Fas-activated serine/threonine (FAST) kinase domain containing protein 5), has been suggested to be required for maturing precursor of COI mRNA (Antonicka and Shoubridge 2015). However, the exact role of these proteins in relation to mtRNA endonucleolytic processing remains to be determined.

### Mitochondrial mRNA maturation and maintenance

#### Mitochondrial mRNA polyadenylation

Mitochondrial mRNAs liberated by mt-tRNA excision undergo further maturation steps, however, the scope of these events is very limited when compared to the nuclear-encoded mRNAs. Mitochondrial mt-mRNAs do not contain the 5′ cap modification nor introns to be spliced. Most mt-mRNAs are polyadenylated on their 3′ ends, with poly(A) tails being much shorter (approximately 45–55 nt) than the ones found on the nDNA-encoded mRNAs. Poly(A) tails have not been detected in the case of ND6 mt-mRNA and the ND5 transcript is oligoadenylated (or not adenylated at all) (Temperley et al 2010).

Mitochondrial poly(A) tails are synthesised by a non-canonical poly(A) polymerase (mtPAP) that localises to the mitochondrial RNA granules (Tomecki et al 2004; Nagaike et al 2005; Bai et al 2011; Wilson et al 2014). Knockdown of mtPAP causes shortening of poly(A) tails and disruption of the respiratory function (Nagaike et al 2005). Although mtPAP can use all four nucleoside triphosphates (NTPs) as substrates, it is most active with ATP (Bai et al 2011).

Phosphodiesterase 12 (PDE12), a mitochondrial 2′- and 3′-phosphodiesterase (Poulsen et al 2011; Rorbach et al 2011), has been shown to specifically remove poly(A) tails from mt-mRNAs in vitro and in cultured cells upon overexpression (Rorbach et al 2011). The 3′-to-5′ deadenylase activity of PDE12 requires a free 3′-OH group and prefers homopolymers of adenines.

The exact role for mitochondrial poly(A) tails is still unclear (Rorbach et al 2014a). However, seven out of 13 mtDNA-derived ORFs do not encode complete stop codons for the termination of translation. In these cases, the mt-mRNAs are immediately adjacent to mt-tRNA genes and the excision of mt-tRNA leaves behind an incomplete stop codon of ‘U’ or ‘UA’ (Anderson et al 1981). Polyadenylation generates a complete ‘UAA’ stop codon (Ojala et al 1981).

Polyadenylation has different effects on the stability of various mitochondrial transcripts. It generally decreases the stability of COI, COII, COIII and ATP6/8, and increases the stability of ND1 and ND2. Polyadenylation also causes the increase in stability of ND3, ND4/4 L, ND5 and CytB to a lesser extent (Rorbach et al 2011; Wydro et al 2010; Rorbach and Minczuk 2012). The mechanism of this transcript-specific role of polyadenylation remains to be elucidated.

Whether poly(A) tail addition is directly involved in regulating mitochondrial translation in the mitochondria is still an unresolved issue. Overexpression of mitochondrial deadenylase PDE12 or mitochondrially targeted cytosolic poly(A)-specific ribonuclease (PARN) caused strong inhibition of protein synthesis. This is further supported by the observation that targeting cytosolic poly(A)-binding protein 1 (PABPC1) into the mitochondria inhibited translation (Wydro et al 2010). However, inactivation of mitochondrial protein LRPPRC (see the next section) in a mouse knockout model or loss-of-function mutations in MTPAP (Wilson et al 2014) (described below) were associated with the loss of mt-mRNA poly(A) tails with no universal effect on translation. The latter result suggests that translation of some mitochondrial transcripts can be effective even in the absence of mt-mRNA polyadenylation.

#### Mitochondrial mRNA stability, decay and surveillance

Regulation of RNA stability and turnover are important steps in post-transcriptional control of gene expression and are usually mediated by protein complexes. Several proteins have been implicated in these processes in human mitochondria.

The human Suv3 protein (hSuv3p, Suppressor of Var1, 3-like protein 1, SUPV3L1) is an NTP-dependent helicase that localises to the mitochondria. The *SUPV3L1* gene undergoes alternative splicing and contains more than one translation initiation site, the latter observed for a number of mitochondrially-targeted proteins (Minczuk et al 2005; Szczesny et al 2007; Kazak et al 2013). At least one of the isoforms localises to the mitochondrial matrix (Minczuk et al 2002). hSuv3p is able to unwind multiple DNA and RNA substrates (Minczuk et al 2002; Shu et al 2004). It has been suggested that hSuv3p is involved in mtRNA degradation of aberrant mtRNA (Khidr et al 2008; Szczesny et al 2010) and plays a role in the decay pathway of properly processed RNA molecules (Szczesny et al 2010).

Polynucleotide phosphorylase, PNPase (encoded by *PNPT1*), is capable of 3′-to-5′ phosphorolysis and 5′-to-3′ polymerisation of RNA (Wang et al 2009). PNPase has been reported to form complexes localised in the matrix, although mitochondrial inner membrane space localisation has also been observed (Chen et al 2006). PNPase has been attributed to numerous roles in mtRNA metabolism, including degradation, polyadenylation and RNA import (Chen et al 2006; Slomovic and Schuster 2008; Borowski et al 2013; Chujo et al 2012).

Recent evidence indicates that PNPase co-localises with hSuv3p in the mitochondria in distinctive RNA-positive foci and works in collaboration to facilitate RNA degradation (Szczesny et al 2010). This suggests that there might be two pools of PNPase, in the intermembrane space and in the matrix (Borowski et al 2013). In vitro reconstitution experiments have indicated that the hSuv3-PNPase complex degrades RNA substrates in the 3′-to-5′ direction (Wang et al 2009; Lin et al 2012).

The RNA exonuclease 2, REXO2, is an additional mitochondrial nuclease potentially involved in mtRNA metabolism. It is a homotetrameric 3′-to-5′ exonuclease that degrades oligonucleotides and is localised to both the intermembrane space and the matrix (Bruni et al 2013). Given that the PNPase-hSuv3p complex is expected to degrade RNA producing oligo-ribonucleotides, it is possible that they form the substrate of REXO2 for later stages of the decay pathway. REXO2 has also been proposed to have a function in recycling mono-ribonucleotides (Bruni et al 2013).

RNA-binding proteins are often key regulators of the post-transcriptional life of transcripts. Two mitochondrial RNA-binding proteins, LRPPRC and SLIRP, have been recently associated with mtRNA metabolism. LRPPRC is a highly abundant 130 kDa leucine-rich PPR containing protein, and is predominately localised in the mitochondrial matrix (Sterky et al 2010). Inactivation of LRPPRC results in a severe depletion of mt-mRNA, but not mt-rRNA or mt-tRNA, and a consequential impairment in translation and in assembly of mitochondrially-encoded OXPHOS subunits (Gohil et al 2010; Sasarman et al 2010a; Sondheimer et al 2010; Ruzzenente et al 2012; Mourier et al 2014). Stem-loop interacting RNA binding protein, SLIRP, is essential for mt-RNA maintenance (Baughman et al 2009) and exists in a high-molecular-weight complex with LRPPRC (Sasarman et al 2010b). The LRPPRC-SLIRP complex regulates mt-mRNA stability and translation without directly associating with ribosomes or POLRMT (Ruzzenente et al 2012; Harmel et al 2013). Knockdown of one of the two proteins caused a decrease in endogenous levels of the other, suggesting strong interdependence (Chujo et al 2012; Ruzzenente et al 2012). The LRPPRC-SLIRP complex has been recently associated with a role in mt-mRNA polyadenylation. Inactivation of either LRPPRC or SLIRP resulted in a reduction in polyadenylated mt-mRNAs, with an in vitro stimulatory effect of LRPPRC-SLIRP, but not SLIRP alone, on polyadenylation by mtPAP (Chujo et al 2012; Wilson et al 2014). It has been suggested that LRPPRC plays a role in stabilising a pool of translationally-inactive mt-mRNAs that are not associated with ribosomes (Ruzzenente et al 2012). The LRPPRC-SLIRP complex may also function by binding to the mt-mRNA and preventing the formation of secondary structures making the 3′-terminal available for polyadenylation. This activity of the complex might also be involved in suppressing PNPase-hSuv3-mediated mt-mRNA degradation (Chujo et al 2012).

### Mitochondrial rRNA maturation

Human mitochondrial ribosomes are composed of two unequally sized subunits (Brown et al 2014; Greber et al 2014; Kaushal et al 2014; Amunts et al 2015). The small 28S subunit (mtSSU) plays a role in catalysing the peptidyl-transferase reaction, while the large 39S subunit (mtLSU) is involved in mt-mRNA binding and decoding. These two subunits are composed of 12S mt-rRNA (mtSSU) and 16S mt-rRNA (mtLSU) and ribosomal proteins. The biogenesis of the mitochondrial ribosome is a complex process entailing a number of auxiliary factors (Rorbach et al 2012; Dalla Rosa et al 2014). Maturation of mt-rRNA through a number of post-transcriptional nucleotide modifications is necessary for correct folding, stability and ribosome assembly (Decatur and Fournier 2002). However, while cytosolic rRNAs have over 200 modifications and bacterial rRNAs over 30, only nine modified nucleotides have been identified in mt-rRNAs (Piekna-Przybylska et al 2008).

#### Maturation of 12S mt-rRNA

**Fig. 4:**
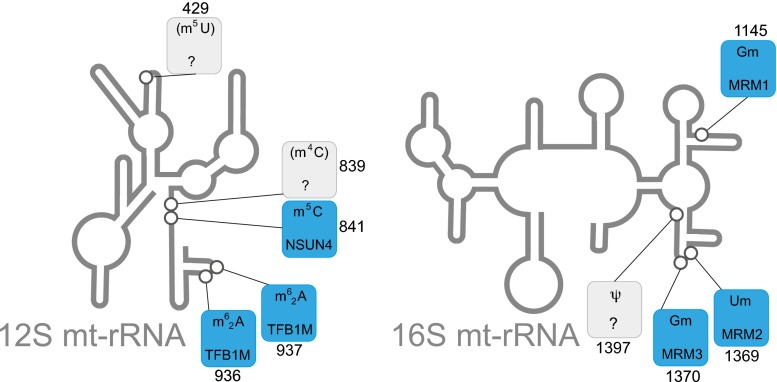
Post-transcriptional modifications of mitochondrial ribosomal RNA. Schematics of the secondary structure of 12S and 16S mt-rRNAs, indicating post-transcriptionally modified bases (circles) is shown. The details of the chemical modification and enzyme responsible (if known) for each mt-rRNA position is given in boxes, indicating the mt-rRNA base position number next to each box. The chemical modifications identified in mammalian species other than human are in brackets. Colour coding: *blue*, enzyme responsible for particular modification has been identified; *grey*, modifying enzyme has not been identified

Mt-tRNA cleavage from the mitochondrial H-strand precursor liberates full-length 12S and 16S mt-rRNAs without a requirement of any further nucleolytic processing events. Five nucleotide modifications have been identified in the mammalian 12S mt-rRNA, with one methylated uracil (12S: m^5^U429, mtDNA position: 1076), two cytosine methylations (12S: m^4^C839 and m^5^C841, mtDNA positions: 1486 and 1488, respectively) and two dimethylated adenosines (12S: m^6^_2_A936 and m^6^_2_A937, mtDNA positions: 1583 and 1584) (Baer and Dubin 1981). Only two of the proteins responsible for these post-transcriptional nucleotide modifications have been characterised to date (Fig. 4).

TFB1M is responsible for dimethylation of two highly conserved adjacent adenines in a stem-loop structure near the 3′ end of the 12S mt-rRNA, m^6^_2_A936 and m^6^_2_A937 (Seidel-Rogol et al 2003; Metodiev et al 2009). In bacteria, this region contains the mRNA decoding centre and the binding site for the large subunit (Xu et al 2008b). The lack of TFB1M causes a dramatic decline of 12S mt-rRNA levels, leading to a deficiency of assembled mtSSU and results in a severe impairment of mitochondrial translation (Metodiev et al 2009).

Another protein that plays a role in 12S mt-rRNA maturation is NSUN4 (NOP2/Sun domain containing family, member 4), which has been suggested to have a dual function. In a complex with MTERF4, it plays a role in ribosome assembly regulation (Camara et al 2011; Spahr et al 2012; Yakubovskaya et al 2012). However, the NSUN4 protein on its own is capable of m^5^C841 methylation of the 12S mt-rRNA. It has been speculated that m^5^C841 is involved in stabilisation of mt-rRNA folding, in cooperation with m^4^C839 methylation. The methyltransferases required for methylation of 12S mt-rRNA positions C481 and U429 remain unidentified (Fig. 4).

#### Maturation of 16S mt-rRNA

Four nucleotides are modified in the mammalian 16S mt-rRNA: two ribose methylations of guanosine (16S: Gm1145 and Gm1370, mtDNA positions: 2815 and 3040), one ribose methylation of uridine (16S Um1369, mtDNA position 3039), and one pseudouridylated base (16S: Psi1397, mtDNA position: 3067) (Dubin and Taylor 1978; Ofengand and Bakin 1997). Mitochondrial rRNA methyltransferase 1, MRM1, is responsible for 2′-*O*-ribose methylation of 16S mt-rRNA position Gm1145 and is present in mitochondrial RNA granules (Lee et al 2013; Lee and Bogenhagen 2014; Rorbach et al 2014b). In yeast mitochondria, this methylation is crucial for the stability of the large ribosomal subunit and for ribosomal function (Sirum-Connolly and Mason 1993), however the role of this modification in human 16S mt-rRNA has not been investigated. Two other methylated nucleotides of the 16S mt-rRNA, Um1369 and Gm1370 are localised in the A-loop, an essential component of the peptidyl transferase centre of the large subunit rRNA and are involved in the interaction with the aminoacyl site of the mt-tRNA (Decatur and Fournier 2002). These sites are modified by MRM2 and MRM3 (also known as RNMTL1), respectively (Lee et al 2013; Lee and Bogenhagen 2014; Rorbach et al 2014a). Both enzymes are localised in mitochondrial RNA granules and are indispensable for correct mitochondrial translation and OXPHOS function. Ineffective translation in MRM2 and MRM3-depleted cells stems from aberrant assembly of mtLSU (Rorbach et al 2014b). It remains to be established, however, whether the presence of MRM2/MRM3 or rather the presence of the actual modification of 16S mt-rRNA is crucial for mitochondrial ribosome biogenesis. The enzyme responsible for pseudouridylation of the 16S mt-rRNA is still unknown (Fig. 4).

### mt-tRNA maturation

The release of mt-tRNAs from a polycistronic primary transcript does not mark the end of their maturation process (Fig. 2). Instead, numerous post-transcriptional modifications are utilised to form the cloverleaf structure resulting in a stable and correctly functioning tRNA (Helm et al 1999). A great range of chemical diversity exists in the modifications found to mt-tRNAs, with bases undergoing methylations, isomerisations, thiolations, formylations, and ribosylations, along with others, by a multitude of nuclear factors. The impact of a tRNA modification can be broadly divided into those that confer structural stability and correct folding, and those that assist proper tRNA function via altering tRNA interaction with other factors.

Whilst many unmodified tRNAs fold into structures that are in approximately the correct conformation (Sampson and Uhlenbeck 1988; Harrington et al 1993), unmodified mitochondrial tRNAs transcribed in vitro have been demonstrated to fold into a range of non-functional secondary structures (Helm et al 1998). Modifications towards the core of the tRNA typically play a role in its structure and stability, often through the modulation of the rigidity of particular domains. For example, saturation of the pyrimidine ring of uracil to form dihydrouridine results in a greater range of conformational flexibility (Dalluge 1996), while the introduction of pseudouridine, through the isomerisation of uridine, acts to increase the structural rigidity of the tRNA (Davis 1995). Base methylation can also influence tRNA structure in a number of ways. For example, the methylation of adenine in position 9 (A9) in human mt-tRNA^Lys^ by the RNase P subcomplex of MRPP1 and MRPP2 (HSD17B10) (Vilardo et al 2012) impacts on tRNA structure. Unmodified mt-tRNA^Lys^ folds into a non-functional extended hairpin structure, which is not recognised by its cognate lysyl-tRNA synthetase (Helm et al 1998; Sissler et al 2004). The introduction of m^1^A9 alone into mt-tRNA^Lys^ is sufficient to favour the formation of the canonical cloverleaf structure by disrupting the A9-U64 Watson-Crick base pair in the extended hairpin (Helm et al 1999). However, the stabilising effects of the *N*^1^-methylation of adenine have also been identified, on the T-loop at position 58, catalysed in human mitochondria by tRNA methyltransferase TRMT61B (Chujo and Suzuki 2012).

Modifications at tRNA residue positions 34 and 37 are important for maintaining translation accuracy and fidelity, representing the greatest chemical diversity found in a tRNA’s modification profile. One or both of these positions are modified in almost all tRNAs so far studied. The translation of an mRNA into its corresponding polypeptide chain is dependent on the precise interactions between the three bases (referred to as 1, 2 and 3) of the mRNA’s triplet codon and the triplet anticodon of the cognate tRNA (at positions 36, 35 and 34). However, owing to degeneracy of the genetic code, multiple codons must be recognised by a single tRNA. Degenerate codons contain identical residues in positions 1 and 2 and are expanded through variability in position 3. To accomplish this, the interactions between residues 3 and 34 are non-standard, allowing for a much greater range of possible base pairs, a characteristic referred to as ‘wobble’. Position 34, or the wobble base, is often occupied by a uridine, capable of base pairing with any of the four bases due to enhanced conformational flexibility within the anticodon loop. This scenario is sufficient for the majority of codons in which the residue in position 3 is entirely degenerate. However, in a number of cases, the presence of a purine or a pyrimidine in position 3 produces codons for different amino acids. The increase in discrimination by the wobble base required for accurate decoding is achieved through its post-transcriptional modification (Johansson et al 2008). For example, the formation of the τm^5^(s^2^)U modification by the concerted action of GTP binding protein 3, GTPBP3, (Villarroya et al 2008), MTO1 (mitochondrial tRNA translation optimisation 1) (Li et al 2002) and MTU1 (mitochondrial tRNA-specific 2-thiouridylase, TRMU) (Yan et al 2006a), greatly favours base pairing with purines and prevents codon misreading (Yokoyama et al 1985) as has been demonstrated for human mt-tRNA^Leu(UUR)^ (Kirino et al 2004). The recognition of purines at position 3 is also exemplified by the formylation in mt-tRNA^Met^, the resulting f^5^C34 allows the tRNA to read the AUA as well as AUG codons (Bilbille et al 2011). Whilst stringent selection of the correct cognate tRNA is key to ensuring accuracy, a stable codon-anticodon interaction is critical for translation efficiency. For this reason, tRNAs with anticodons bearing U and A in position 36 often require modification at the adjacent position 37 (Lamichhane et al 2013a). For example, in mammalian mitochondria position 37 contains *N*^6^-threonylcarbamoyladenosine (t^6^A37) and *N*6-isopentenyladenosine (i^6^A37), catalysed by TRIT1 (tRNA isopentenyltransferase 1) in human cells (Yarham et al 2014) and further modified by CDK5RAP1 (Cyclin-dependent kinase 5 regulatory subunit associated protein 1) to form ms^2^i^6^A37 (Reiter et al 2012). A guanosine residue may also be present at position 37 in human mt-tRNAs, which is often methylated to form *N*^1^-methylguanosine (m^1^G37) by TRMT5 (Brule et al 2004). The m^1^G modification disrupts the formation of Watson-Crick base pairing, therefore blocking potential base pairing between G37 and the mRNA to prevent frameshifting (Urbonavicius et al 2001; Urbonavicius 2003). By interfering with base pairing, m^1^G37 may also prevent interactions with neighbouring nucleotides on the other side of the anticodon loop to aid in the formation of the canonical loop structure required for codon-anticodon interactions (Cabello-Villegas et al 2002).

Lastly, post-transcriptional modifications of tRNAs may assist in translational fidelity through ensuring correct aminoacylation. The recognition of a tRNA by its cognate tRNA-aminoacyl synthetase depends on the identification of particular nucleotides and structural motifs, predominantly within the anticodon loop and the acceptor helix, such as the discriminator base at position 73, and the 5′ terminal CCA added post-transcriptionally by TRNT1 (Nagaike et al 2001). The majority of modifications are assumed to contribute to tRNA identity indirectly through the stabilisation of a recognised structural feature. However, a direct identity element role for a modified nucleotide, either as a recognition determinant as in Ψ35 in yeast tRNA^Tyr^ (Bare and Uhlenbeck 1986), or an antideterminant as has been described for m^1^G37 in yeast tRNA^Asp^ (Putz et al 1994), cannot be ruled out in human mitochondrial tRNAs.

**Fig. 5:**
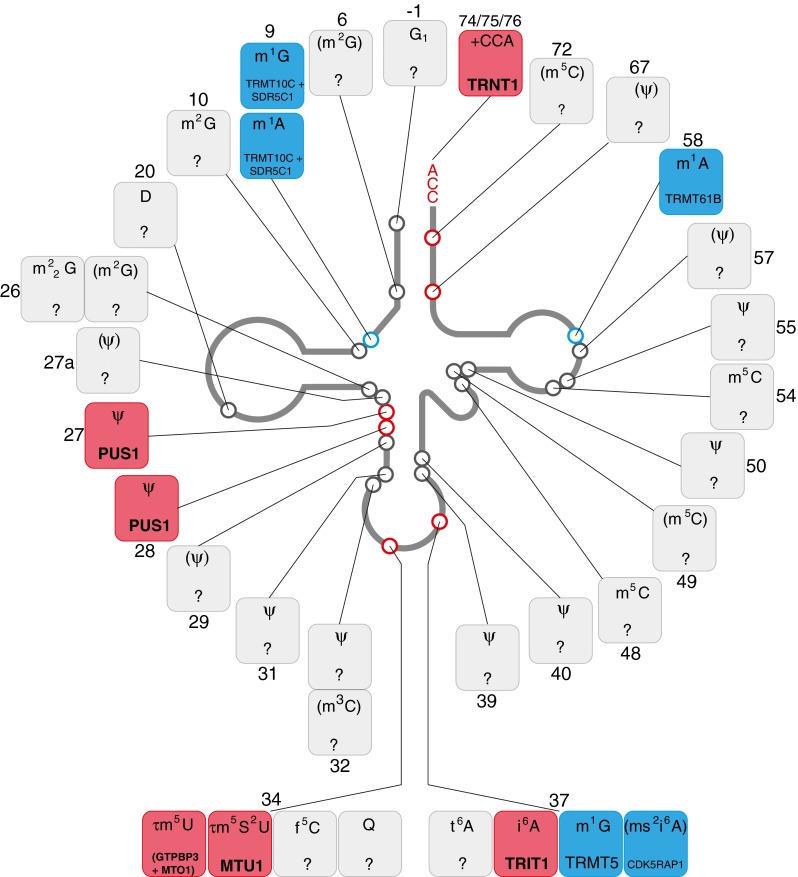
Post-transcriptional modifications of mitochondrial transfer RNA and human disease. Schematics of the “clover leaf” secondary structure of a generic mitochondrial tRNA indicating post-transcriptionally modified bases (circles) is shown. The details of the chemical modification and the enzyme responsible (if known) for each mt-tRNA position is given in boxes, indicating the mt-tRNA base position number next to each box. The chemical modifications identified in mammalian species other than human are in brackets. Colour coding: *red*, enzyme responsible for the modification has been associated with human disease; *blue*, enzyme responsible for particular modification has been identified; *grey*, modifying enzyme has not been identified

A complete set of modifications for bovine mt-tRNAs, which share a high degree of structural and sequence similarity with human mt-tRNAs, have recently been described (Suzuki and Suzuki 2014). From this work, many human mt-tRNA modifying enzymes may be predicted, bringing us closer to a comprehensive modification profile for human mt-tRNAs (current status summarised in Fig. 5, expanded upon in Powell et al (2015) and Suzuki et al (2011).

## Human diseases associated with defects of mitochondrial RNA maturation

### Defects of mt-pre-RNA processing

#### HSD17B10

As discussed in Nucleolytic processing of mitochondrial precursor RNA, the mitochondrial RNase P complex consists of three protein components, MRPP1, 2 and 3. The exact role of MRPP2, also known as 17β-hydroxysteroid dehydrogenase type 10 (HSD17B10), in the RNase P complex is unknown. However, it is a multifunctional mitochondrial protein which, in addition to its functions in mtRNA maturation, catalyzes the 2-methyl-3-hydroxylbutyryl-CoA dehydrogenation (MHBD) reaction in isoleucine metabolism, converting 2-methyl-3-hydroxybutyryl-CoA to 2-methyl-acetoacetyl-CoA, as well as playing a potential role in the pathogenesis of Alzheimer’s disease (Chen and Yan 2007) (Table 1).

**Table 1:** Mitochondrial transcript metabolism in human disease

	Gene*	MIM	Function	Target*	Disease	References
Precursor transcript processing						
	*MRPP2* (*HSD17B10*)	300256	Endonucleolytic processing (5′ end of mt-tRNA)	Precursor mt-RNA	Neurological abnormalities and cardiomyopathy	Deutschmann et al 2014
	*ELAC2*	605367	Endonucleolytic processing (3′ end of mt-tRNA)	Precursor mt-RNA	Infantile hypertrophic cardiomyopathy and complex I deficiency	Haack et al 2013
Transcript polyadenylation, stability & turnover						
	*LRPPRC*	607544	Post-transcriptional regulation?	mt-mRNA	Leigh syndrome French Canadian type (LSFC)	Morin et al 1993; Mootha et al 2003
	*PNPT1*	610316	RNA degradation (or RNA import)	?	Encephalomyopathy, deafness	Vedrenne et al 2012; von Ameln et al 2012
	*MTPAP*	613669	mt-mRNA polyadenylation	mt-mRNA	Spastic ataxia	Crosby et al 2010; Wilson et al 2014
mt-tRNA modification						
	*TRNT1*	612907	Addition of 3′ CCA		Sideroblastic anemia with immunodeficiency, fevers and developmental delay (SIFD)	Chakraborty et al 2014
	*PUS1*	608109	Ψ27, Ψ28	mt-tRNA: Asp, Ile, Lys, Met, Leu(CUN), Leu(UUR), Cys, His, Glu, Asn, Tyr	Mitochondrial myopathy lactic acidosis and sideroblastic anaemia	Patton et al 2005; Zeharia et al 2005; Fernandez-Vizarra et al 2007
	*GTPBP3*	608536	τm^5^U34	mt-tRNA: Lys, Leu(UUR), Trp, Gln, Glu	Hypertrophic cardiomyopathy, lactic acidosis and encephalopathy	Kopajtich et al 2014
	*MTO1*	614667	τm^5^U34	mt-tRNA: Lys, Leu(UUR), Trp, Gln, Glu	Hypertrophic cardiomyopathy and lactic acidosis	Ghezzi et al 2012; Baruffini et al 2013
	*MTU1* (*TRMU*)	610230	τm^5^s^2^U34	mt-tRNA: Lys, Gln, Glu	Acute infantile liver failure/mitochondrial-associated deafness	Zeharia et al 2009; Schara et al 2011; Uusimaa et al 2011; Gaignard et al 2013
	*TRIT1*	–	i^6^A37	mt-tRNA:Ser(UCN), Cys, Phe, Trp, Tyr	Encephalopathy and myoclonic epilepsy	Yarham et al 2014
	MTFMT**	611766	Formylmethionine	mt-tRNA: Met	Leigh Syndrome	Tucker et al 2011; Neeve et al 2013; Haack et al 2014

List of nuclear-encoded disease-causing genes involved in precursor transcript processing, mt-mRNA transcript polyadenylation, stability and turnover and mt-tRNA modification. Mt-tRNA species with confirmed nucleotide modification in human are underlined. The other modifications are predicted based on the analysis of bovine mt-tRNAs (Suzuki and Suzuki 2014)

** MTFMT is strictly speaking a modifier of a charged amino acid, rather than of the mt-tRNA itself

Patients in at least 19 independent families have been reported to carry pathogenic, X-linked mutations in HSD17B10 [MIM: 300256] (HSD17B10 is located at Xp11.2) (Zschocke 2012). Affected patients suffer from a progressive loss of cognitive and motor function, epilepsy, retinal degeneration and progressive cardiomyopathy, with lactic acidosis also a common presentation. Infantile onset is most common, with onset of symptoms following the first 6–18 months of life, resulting in death at age 2–4 years or later. A more severe neonatal form has been characterised with severe progressive cardiomyopathy with limited neurological presentation, resulting in early death (Perez-Cerda et al 2005). Due to the X-linked nature of inheritance, symptoms have been shown to vary between male and female patients carrying the same mutation in HSD17B10 (Perez-Cerda et al 2005).

The severity of clinical symptoms presenting in patients with mutations in HSD17B10 are suspected to be independent of relative residual MHBD activity in different mutant variant proteins (Rauschenberger et al 2010), and attempts at placing patients on isoleucine restricted diets have had mixed success at preventing further clinical deterioration (Zschocke et al 2000; Perez-Cerda et al 2005). Additionally, the neurodegenerative disorder experienced in patients with HSD17B10 deficiency is dissimilar to other disorders of isoleucine metabolism or other organic acidurias, and more closely resembles a primary mitochondrial disorder (Rauschenberger et al 2010). This evidence may suggest that the deficiency in MHBD activity of HSD17B10 may not be the primary cause of clinical presentation of the disease, and is instead linked to an independent mitochondrial dysfunction.

In a recent article, Deutschmann et al (2014) have suggested that the disorder associated with mutations in HSD17B10 may instead be linked to the role of HSD17B10 (MRPP2) in mtRNA endonucleolytic processing. In this study, patient fibroblasts harbouring a p.R130C mutation in HSD17B10, which causes infantile onset disease, were shown to have a lower residual protein level of HSD17B10 compared to controls, which was accompanied by a reduction in MRRP1 protein, but not MRPP3. The study showed there was impaired processing of the H-strand precursor mtRNA transcript, but not the L-strand, compared to controls. This deficiency in mtRNA processing may contribute to the mitochondrial disorder experienced in HSD17B10 patients.

Due to the multifunctional nature of the protein, it is possible that mutants at different sites within HSD17B10 may have independent effects on mtRNA processing and MHBD activity, which in turn may influence the clinical presentation and severity of the disorder.

#### ELAC2

Pathogenic mutations in the *ELAC2* gene, which encodes the nuclease that cleaves 3′ off mt-tRNAs, have recently been identified [MIM: 605367] (Haack et al 2013). Initial exome sequencing of two unrelated probands who each exhibited a suspected infantile-onset mitochondrial disorder identified mutations in *ELAC2* as the possible underlying defect. Further targeted sequencing of *ELAC2* in 350 individuals with oxidative phosphorylation disorders identified two additional cases of genetic variation in *ELAC2* in siblings of an unrelated family. In this study, a total of five individuals were identified across three independent families, harbouring either compound heterozygous or homozygous mutations in ELAC2.

Like many mitochondrial disorders, severity of disease progression varied among individuals, but all patients presented with some consistent clinical features. All individuals presented with poor growth, onset of infantile hypertrophic cardiomyopathy prior to 6 months and elevated lactate levels and associated (or consequent) alanine and occasional increase of glutamine and ammonia, consistent with a status of metabolic acidosis. Isolated complex I deficiency in skeletal muscle was observed in all patients, with one individual also exhibiting a complex IV deficiency. In four out of five individuals studied, a mild to marked developmental delay was also apparent. Two patients died before 12 months of age and a third died at the age of 4 years.

If variants in ELAC2 were indeed the underlying primary defect, it was expected that processing at sites 3′ to mt-tRNAs would be hindered due to lack of ELAC2 activity. All tRNA-mRNA junctions expected to be cleaved by the ELAC2 protein showed accumulation in patient skeletal muscle, and, to a lesser extent, in patient fibroblasts, as assessed by qPCR. This was confirmed by RNA-Seq and northern blotting, consistent with the characterised RNase Z activity of ELAC2 in mitochondria. Notably, changes to steady state levels of correctly processed mt-tRNAs, mt-rRNAs and mt-mRNAs were not observed, despite the accumulation of unprocessed intermediates.

A further individual, suffering from a mitochondrial disorder, was characterised as having a presumed pathogenic mutation in *ELAC2*. This patient was identified recently via exome sequencing of 53 patients carrying multiple respiratory chain complex defects (Taylor et al 2014). In the same high-throughput-exome sequencing study a single individual was identified as carrying possible pathogenic mutations in the PTCD1 gene, the protein product of which is thought to directly interact with ELAC2. However, as only a single individual has so far been identified, and mutations in PTCD1 have not previously been associated with a mitochondrial disorder, further investigation will be required to confirm PTCD1 as the causative gene in this case.

### Defects of mt-mRNA maturation

#### PNPase

Three studies have identified pathogenic mutations in the *PNPT1* gene, encoding PNPase, thought to be involved in mtRNA turnover and possibly RNA import into mitochondria, as earlier described.

The first study, by Vedrenne et al (2012), identified two siblings, born to first-cousin healthy Moroccan parents, suffering with severe but non-progressive encephalopathy accompanied with elevated plasma and cerebrospinal-fluid lactate levels [MIM: 614932]. Affected individuals developed severe hypotonia and movement abnormalities in late infancy. Exome sequencing of the siblings identified a homozygous c.1160A > G variant in the *PNPT1* gene in both individuals. This variant produces a p.Gln387Arg transition in the PNPase protein product, with the affected glutamine residue lying within a highly conserved domain of the protein. Mitochondrial translation was shown to be moderately reduced in patient fibroblasts, which is rescued upon reintroduction of wild-type PNPase cDNA, supporting the argument that the variant in *PNPT1* is the underlying primary cause of the mitochondrial disorder.

The second study also concerned a single consanguineous Moroccan family, where three siblings were affected by a severe, early onset hearing impairment in early childhood [MIM: 614934] (von Ameln et al 2012). Here, it was shown via genome-wide homozygosity mapping that the three affected siblings carried a homozygous c.1424A > G mutant variant (p.Glu475Gly) in *PNPT1* which co-segregated on the pedigree with the hearing loss.

Recently, an individual in a third suspected family was shown to carry compound heterozygous sequence variants in the *PNPT1* gene (Slavotinek et al 2014), a maternally inherited mutation affecting the acceptor splice-site for exon 5, c.401-1G > A, and a paternally inherited c.1519G > T missense mutation (p.Ala507Ser). In this case, as well as clinical presentations, which are consistent with the previous reports (microcephaly, myoclonic epilepsy and sensorineural deafness), the proband also suffers from eye manifestations in the form of left-sided peripheral chorioretinal defect and optic atrophy (Slavotinek et al 2014).

The difference in severity and tissue specificity in the individuals affected across the three studies may reflect a different level of residual PNPase activity for the different mutant variant proteins. This example highlights the heterogeneity of the presentation of mitochondrial disorders.

In the first two reported cases it was shown that the mutations affected homo-trimerisation of PNPase. To determine the exact mechanism perturbed in the disease, both studies focused on the deleterious effect of the mutant PNPase variants on the reported role of PNPase in RNA import, where it was shown that both mutant variants resulted in impaired import of RNAs into mitochondria. However, the exact role of PNPase in human mitochondria is still a subject of debate, and it had not been addressed whether either of the mutant proteins had any effect on the role of PNPase in the mitochondrial matrix, where it has been shown to be involved in mtRNA turnover (Borowski et al 2013).

#### mtPAP

Six individuals from a large consanguineous Old Order Amish family were reported to carry a homozygous pathogenic mutation in *MTPAP*. Patients suffer from a slowly progressive autosomal-recessive neurodegenerative disorder characterised by spastic paraparesis, cerebellar ataxia and optic atrophy [MIM: 613669] (Crosby et al 2010). This is also often accompanied with learning difficulties.

Genome wide linkage and homozygosity mapping of affected individuals identified a region of 6.5 Mb at chromosome 10p11.23 as likely to contain the disease locus. Targeted sequencing of genes within this region identified a homozygous c.1432A > G mutation in *MTPAP* which cosegregated with the disease phenotype. The mutation results in a predicted p.N478D substitution in a highly conserved region of mtPAP protein.

Mitochondrial poly(A) tail assays using RNA from blood of affected patients revealed a profound reduction of polyadenylation of several mtRNAs in homozygous carriers (Crosby et al 2010), although transcripts did remain oligoadenylated (<10 nt 3′ extension), and a further study was able to confirm that the p.N478D mutant mtPAP retained residual oligoadenylation activity, but not polyadenylation activity (Wilson et al 2014). This highlights polyadenylation of mitochondrial mRNAs as essential for mitochondrial gene expression, as reduction of polyadenylation due to impaired mtPAP activity was later shown to cause a mitochondrial translation defect (Wilson et al 2014).

#### LRPPRC

Leigh syndrome French Canadian (LSFC) type [MIM: 220111] is a severe autosomal recessive disorder which presents in the geographically isolated Saguenay-Lac-Saint-Jean region of Quebec, resulting from mutations in the LRPPRC gene. In this region, it is estimated that ~1 in 23 adults are carriers of the affected mutant LRPPRC allele, resulting in LSFC presenting in around 1 in 2000 births (Morin et al 1993). Genealogical analysis suggests the LRPPRC founder mutation to originate from individuals of the French-Canadian population in Europe in the 17th Century. This founder allele carries a p.A354V mutation at a highly conserved residue of the LRPPRC protein.

LSFC is characterised by severe COX deficiency, which particularly affects the liver and brain, and to a lesser extent in fibroblasts and skeletal muscle, due to a failure to assemble the holoenzyme complex (Merante et al 1993). As with classical Leigh syndrome, affected LSFC patients suffered from a severe neurological disorder characterised by subacute necrotising encephalopathy, but also presented with microvesicular steatosis (Morin et al 1993), likely due to the low levels of COX activity in the liver of patients (Merante et al 1993). Other common presentations include a moderate developmental delay, hypotonia, ataxia, strabismus, opthalmoplegia, optic atrophy and mild facial dysmorphia. Patients suffer from elevated lactate levels in blood and cerebrospinal fluid, and most affected individuals died of fulminant metabolic acidosis, at an average age of between 3 and 5 years (Morin et al 1993).

At a stage when whole exome sequencing was not yet available, the affected genomic locus was narrowed down using genome-wide linkage disequilibrium mapping to a ~2 cM region at 2p16-21 (Lee et al 2001). An integrative genomics approach, where the combination of available genomic DNA data, identification of mRNAs which co-express with known mitochondrial genes and proteomic analysis of mitochondrial proteins, was further used to identify LRPPRC as the affected mitochondrial gene in LSFC (Mootha et al 2003). In this study, 21 out of 22 patients carried homozygous founder mutation alleles, with the remaining patient a compound heterozygote.

In patients fibroblasts, a reduction in level of the mutant LRPPRC protein in mitochondria was associated with a specific reduction in steady state levels of COI and COIII mRNAs, and an accompanied specific reduction in COI and COIII translation products (Xu et al 2004), which could explain the specific COX deficiency in LSFC patients (Merante et al 1993). This would suggest that LRPPRC has a specific mRNA stabilising function for COI and COIII mRNAs.

Recently, it has been proposed that tissue specific differences in the levels of LRPPRC and its interacting partner SLIRP in affected tissues may account for the tissue specific biochemical deficiencies encountered in LSFC. While there is an almost complete lack of complex IV in the liver, there is only a 50 % reduction of COX assembly in the heart (Sasarman et al 2015a).

In unaffected control tissues, fibroblasts exhibited the highest levels of the LRPPRC-SLIRP protein complex, with lower levels in muscle and liver, and almost undetectable levels in control heart tissue (Sasarman et al 2015b). This contrasts with levels of assembled complex IV in these control tissues, where heart carries the highest level of complex IV, and liver and fibroblasts have a reduced level. This may suggest a tissue-specific post-transcriptional handling of mitochondrial mRNAs, where heart tissue may be less dependent on activity of the LRPPRC-SLIRP complex compared to heart and brain, which are the main affected tissues when LRPPRC activity is reduced in LFSC.

Additionally, further depletion of LRPPRC protein levels in patient myoblasts and differentiated myotubes, which suffer from complex I as well as complex IV deficiency, leads to a general OXPHOS complex deficiency in all complexes with subunit contributions from mtDNA (Sasarman et al 2015a). It is possible therefore that while the p.A354V founder mutation protein specifically affects COX transcripts and therefore has a greatest effect on cytochrome oxidase assembly, alternative mutations which confer further loss of residual LRPPRC activity may lead to a more generalised combined OXPHOS deficiency.

### Defects of mt-rRNA maturation

Most enzymes that play a role in mt-rRNA maturation have been discovered only recently and very little is known about their possible pathological role. Mouse models exist for only two of these genes (TFB1M and NSUN4) and for both of them, homozygous gene ablation is embryonic lethal. It has been suggested that the function of TFB1M as 12S mt-rRNA methylase might be related to aminoglycoside antibiotics induced deafness (Raimundo et al 2012). However, recent methylation analysis in patients harbouring mtDNA mutation that predisposes to hearing loss following aminoglycoside antibiotic exposure show no difference in 12S mt-rRNA methylation level compared to controls (O'Sullivan et al 2015).

### Defects of mitochondrial tRNA maturation

#### TRNT1

The universally conserved CCA sequence is found on the 3′ terminus of all tRNAs. However, it is uncoded in almost all species, with a post-transcriptional polymerisation event required to produce the full length tRNA. Once mature, the newly polymerised 3′ terminus acts as the amino acid attachment site for the tRNAs cognate aminoacyl-tRNA synthetase. In human mitochondria, this sequence is created by tRNA-nucleotidyltransferase 1 (TRNT1) (Fig. 5) (MIM: 612907). A cohort of patients presenting with SIFD (Sideroblastic anaemia associated with immunodeficiency, periodic fevers and developmental delay) were identified as carrying mutations in TRNT1 (Chakraborty et al 2014). Sideroblastic anaemia, a condition characterised by a reduced ability to incorporate iron into haemoglobin, has been linked to deficiencies in haem synthesis, mitochondrial iron-sulphur cluster biogenesis and mitochondrial translation (Fleming 2011). Some patients were also found to have sensorineural hearing loss, cardiomyopathy and central nervous system abnormalities. All but one of the mutations described were found to have diminished polymerisation activity in an in vitro assay, with the degree of the enzymatic impairment found to correlate with the range of clinical severity found. More recently, two further individuals carrying distinct TRNT1 mutations have been characterised and shown to exhibit an impairment of CCA addition specific to mt-tRNA^Ser(AGY)^ (Sasarman et al 2015b). Despite this, the two individuals presented with varied clinical features: acute lactic acidosis, severe developmental delay, hypotonia, microcephaly, seizures, progressive cortical atrophy, neurosensorial deafness, sideroblastic anaemia and renal Fanconi syndrome in one case, gait ataxia, dysarthria, gross motor regression, hypotonia, ptosis and ophthalmoplegia in the other.

#### PUS1

Given the stabilising role of pseudouridine (Ψ), its absence in mt-tRNAs would be expected to negatively impact translation through the disruption of structure dependent processes, such as its recognition by a cognate aminoacyl-tRNA synthetase, or its accommodation into the P or A site of the ribosome. Of the 13 pseudouridine synthases identified in humans, PUS1 is by far the most well characterised, with loss of function mutations in PUS1 resulting in diminished levels of Ψ27 and Ψ28 in human mitochondrial tRNAs (MIM: 609109) (Patton et al 2005) (Fig. 5). Patients with mitochondrial myopathy, lactic acidosis and sideroblastic anaemia (MLASA), along with a concordant decrease in mitochondrial translation rate, have been identified as carrying causative mutations in PUS1 (Bykhovskaya et al 2004a; Fernandez-Vizarra et al 2007). Despite its dual localisation, with substrates including both cytosolic and mitochondrial tRNAs, patients with deleterious mutations in PUS1 have a characteristic mitochondrial phenotype. The relative sparseness of modifications in mitochondrial tRNAs may result in a greater susceptibility compared to their cytosolic counterparts. For example, a stabilising Ψ55 modification that is present in cytosolic tRNAs is frequently absent in mitochondrial tRNAs. Interestingly, Pus1p, the yeast homolog of PUS1 is found to be non-essential for viability, but is lethal in combination with mutations in Pus4p, the tRNA Ψ55 synthase (Grosshans et al 2001).

#### GTPBP3/MTO1

The incorporation of taurinomethyl at position 34 to form 5-taurinomethyluridine (τm^5^U) has been identified in five mammalian mt-tRNAs: mt-tRNA^Leu(UUR)^, mt-tRNA^Trp^, mt-tRNA^Lys^, mt-tRNA^Gln^ and mt-tRNA^Glu^ (Suzuki and Suzuki 2014). The enzymatic activity responsible for the formation of 5-taurinomethyluridine (τm^5^U) has yet to be definitively demonstrated in humans, however GTPBP3 (MIM: 608536) and MTO1 (MIM: 614667), acting in unison, represent the most likely candidates. Both GTPBP3 and MTO1 have been localised to the mitochondria, and are capable of complementing the mitochondrial phenotypes in yeast strains carrying deletions in their corresponding homologs, MSS1 and MTO1 (Li and Guan 2002; Li et al 2002). Patients presenting with hypertrophic cardiomyopathy, lactic acidosis and a combined OXPHOS deficiency have been found to carry mutations in GTPBP3 (Kopajtich et al 2014) and MTO1 (Ghezzi et al 2012; Baruffini et al 2013). The high degree of similarity of clinical phenotypes between patients with GTPBP3 and MTO1 mutations, an uncharacteristic attribute of mitochondrial disease causing genes, corroborates the notion that these two enzymes act in tandem. The phenotype of an MTO1-deficient mouse model is found to accurately reflect that of the above patients (Becker et al 2014), the severity of which is found to be ameliorated by a ketogenic diet (Tischner et al 2015). Additionally, both GTPBP3 and MTO1 have been identified through linkage analysis as modifier genes in non-syndromic deafness caused by a m.1555A > G mutation in 12S mt-rRNA (Bykhovskaya et al 2004b). Despite being present as a homoplasmic population, the m.1555A > G mutation results in a broad range of clinical severity from individual to individual, with this variance being modulated by the co-inheritance of nuclear-encoded modifier genes (Bykhovskaya et al 1998). Corroboratively, yeast strains bearing a 15S rRNA mutation in the corresponding mtDNA position to human m.1555A > G are found to result in a respiratory defect only in conjunction with mutations in MSS1 and MTO1, yeast orthologous of human GTPBP3 and MTO1, respectively (Decoster et al 1993; Colby et al 1998).

#### MTU1 (TRMU)

Three of the five mt-tRNAs identified as putative GTPBP3/MTO1 substrates carry a more extensively modified 5-taurinomethyl-2-thiouridine (τm^5^s^2^U) wobble base in their mature forms: mt-tRNA^Lys^, mt-tRNA^Gln^ and mt-tRNA^Glu^ (Suzuki et al 2002). As for GTPBP3 and MTO1, yeast deletion strains have greatly assisted in identifying the responsible enzymatic activity, with the loss of the thiouridylase MTU1 (MTO2) resulting in diminished 2-thiolation of mitochondrial tRNAs, with a corresponding impairment of mitochondrial translation. Depletion of the human homolog, MTU1 (MIM: 610230), also known as TRMU, a mitochondrially localised protein capable of complementing yeast MTU1, also produces a dramatic reduction in tRNA thiolation (Umeda et al 2005). Numerous patients with reversible infantile respiratory chain deficiency, a condition characterised by acute liver failure in infancy, have now been identified as carrying causative mutations in MTU1 (Zeharia et al 2009; Schara et al 2011; Uusimaa et al 2011; Gaignard et al 2013). The hypomodification of substrate tRNAs was not found to result in a significant loss of stability, therefore the resulting defective mitochondrial translation is believed to be due to their impaired decoding capabilities (Zeharia et al 2009). Patients present during early infancy with symptoms of liver dysfunction, however, if they survive the first few months of life, make a full spontaneous recovery. It is postulated that infants may experience a window of vulnerability during neonatal development due to a limited availability of sulphur. MTU1 is predicted to be dependent on the activity of a cysteine desulfurase, NFS1, to transfer sulphur from cysteine (Nakai et al 2004), an amino acid that is described as conditionally essential, at least in early life (Zlotkin and Anderson 1982; Zlotkin and Cherian 1988). Cysteine supplementation during this period has therefore been proposed as a potential treatment (Boczonadi et al 2013). As with GTPBP3 and MTO1 above, MTU1 has also been implicated as a modifier gene in mitochondrial deafness (Yan et al 2006b).

#### TRIT1

The existence of the i^6^A37 modification has been demonstrated to improve translation efficiency through the stabilisation of cognate codons, with its loss in yeast, due to deletion of the *tit1* gene, resulting in mitochondrial dysfunction and slow growth (Lamichhane et al 2013b). The homologous isopentyltransferase in humans, TRIT1, was initially identified as a tumour suppressor gene (Spinola et al 2005) with certain variants being associated with lung cancer progression. Subsequent siRNA-mediated TRIT1 depletion studies demonstrated diminished i^6^A37 in cytosolic and mitochondrial tRNAs (Lamichhane et al 2013a). Siblings with encephalopathy and myoclonic epilepsy have been identified as carrying causative mutations in TRIT1 with a corresponding loss of the i^6^A modification and compromised mitochondrial translation (Yarham et al 2014).

#### MTFMT

As in all translation systems, polypeptide synthesis in mitochondria is initiated with a methionine residue. However, metazoan mitochondria differ in that only a single methionine tRNA is utilised for both initiation and elongation. Segregation instead occurs via a post-transcriptional modification, with the initiator mt-tRNA^Met^ undergoing an unusual formylation on the methionine residue charged to its 3′ end, thereby increasing its affinity for the mitochondrial initiation factor (IF2_mt_) (Spencer and Spremulli 2004). Deficiencies in mt-tRNA^Met^-formylation due to mutations in mitochondrial methionyl-tRNA formyltransferase (MTFMT, MIM: 611766) have been identified in a number of patients with Leigh syndrome and combined OXPHOS deficiency (Neeve et al 2013; Haack et al 2014). Significantly reduced mitochondrial translation rates have been identified as the result of pathogenic MTFMT mutations (Tucker et al 2011) and a severely diminished activity of these variants has been demonstrated in vitro (Sinha et al 2014).

#### Mt-tRNA aminoacyl synthetases

The key role of mitochondrial aminoacyl-tRNA synthetases (mtARSs) in human pathology affecting mitochondrial translation has been well recognised. However, this large group of disease genes is not covered by this review. Existing recent papers extensively describe the mitochondrial pathologies caused by genetic defects in ARSs (Konovalova and Tyynismaa 2013; Diodato et al 2014; Vanlander et al 2014).

### Maturation defects related to primary mtDNA mutations

Primary mutations in mtDNA can affect almost any step of mtRNA maturation. Below we provide an overview of pathological mtDNA mutations for which involvement in mt-tRNA, mt-rRNA and mt-mRNA maturation by nuclear encoded genes has been documented.

There are two main types of mtDNA alterations: point mutations, which involve a change of one or a few nucleotides, and large-scale rearrangements, such as large deletions. Unlike the nuclear genome, there are multiple mitochondrial genomes per cell. Therefore, unlike nuclear mutations, which can be either heterozygous or homozygous, mtDNA mutations may be present at any fraction, a condition referred to as heteroplasmy. Cellular physiology is affected only if the mutation fraction in a given cell exceeds a certain threshold, typically >70–90 %, depending on the type of variant and tissues affected. The percentage of mutant mtDNA may vary among individuals and among organs and tissues within the same patient. This, in part, explains the varied clinical phenotype seen in individuals with pathogenic mtDNA mutations (Sciacco et al 1994; Chinnery et al 1997). To complicate this issue even further, one particular base substitution can cause different symptoms in different patients, while a particular syndrome can usually be caused by several possible mutant variants and patients can even evolve from one disease phenotype into another (Schapira 2006). It falls outside the scope of this review to discuss all pathogenic variations.

#### Primary mutations interfering with mt-mRNA maturation

A primary mtDNA mutation interfering with mt-mRNA maturation has been described in two reports. An m.9205ΔTA microdeletion, on the boundary of two genes, ATP6 and COIII has been reported to underlie cases of seizures and lactic acidosis. Encephalopathy has been observed in only one patient (Seneca et al 1996; Jesina et al 2004). Although the two unrelated patients have identical, nearly homoplasmic mutation levels, biochemical consequences of this microdeletion appear to be very different. One patient had no altered primary transcript processing, but a rapid deadenylation leading to enhanced mt-mRNA decay (Seneca et al 1996; Temperley et al 2003), while the other patient showed affected processing of the primary ATP8/6-COIII transcripts, without polyadenylation impairment (Jesina et al 2004).

#### Primary mutations interfering with mt-rRNA maturation

**Fig. 6:**
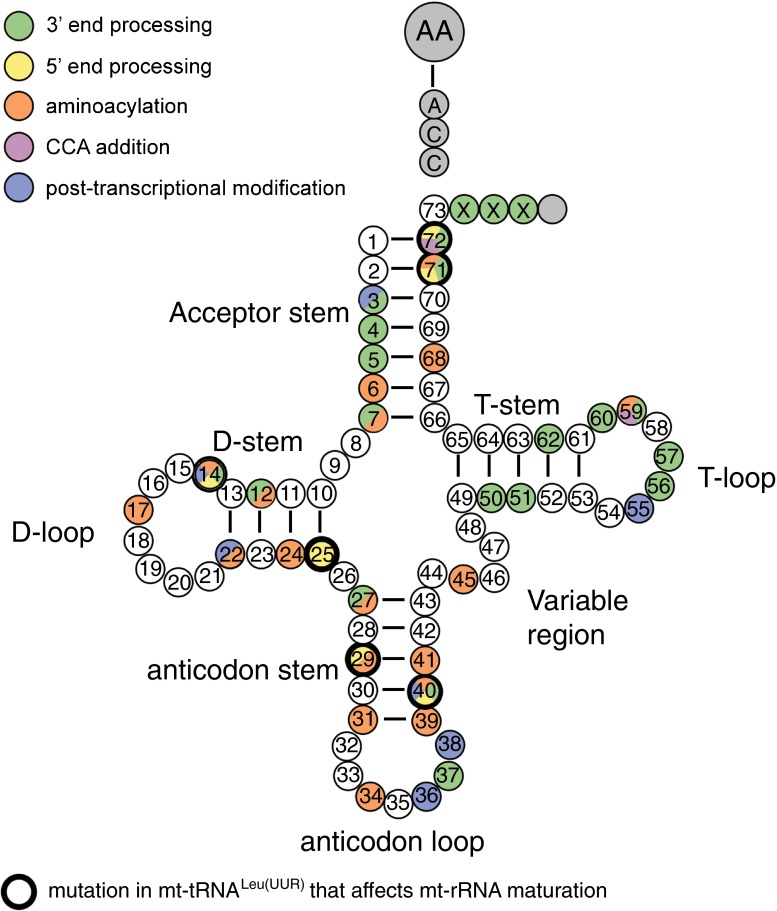
Primary mtDNA mutation affecting mitochondrial RNA maturation. Schematics of the “clover leaf” secondary structure of a generic mitochondrial tRNA indicating the individual positions for which mutations that affect mt-tRNA maturation are described in one or multiple mt-tRNAs (see also Table 2). The “bold circles” show mutations in mt-tRNA^Leu(UUR)^ that affect mt-rRNA maturation as well as mt-tRNA maturation

Point mutations that interfere with mt-rRNA maturation are actually alterations in mt-tRNA^Leu(UUR)^, not in mt-rRNA itself (Fig. 6, bold circles). The common m.A3243A > G MELAS (mitochondrial encephalomyopathy, lactic acidosis and stroke-like episodes) mutation (King et al 1992), as well as m.3271 T > C, m.3303C > T (Koga et al 2003), m.3302A > G (Bindoff et al 1993), m.3260A > G and m.3256C > T (Rossmanith and Karwan 1998), all interfere with end processing of mt-tRNA^Leu(UUR)^ . This causes an accumulation of a precursor RNA consisting of 16S mt-rRNA linked to mt-tRNA^Leu(UUR)^ and ND1 (also known as RNA19) (Rossmanith and Karwan 1998). Although these mutations seem to interfere with 16S mt-rRNA maturation it is presumably not the crucial disease-causing molecular pathway in patients (see below, Table 2).

**Table 2:** Primary mutations in mtDNA that affect mt-tRNA maturation

Affected gene	Mutation	Location	Position	Molecular effect on mt-tRNA maturation	Associated phenotypes	References
mt-tRNA^Phe^	m.T582T > C	Acc-stem	6	aminoacylation	MM	Ling et al 2007
	m.G583G > A	Acc-stem	7	aminoacylation	MELAS / MM & EXIT	Ling et al 2007
	m.A606A > G	AC-stem	29	aminoacylation	Myoglobinuria	Ling et al 2007
	m.A608A > G	AC-stem	31	aminoacylation	Tubulo-interstitial nephritis	Ling et al 2007
	m.G611G > A	AC-loop	34	aminoacylation	MERRF	Ling et al 2007
	m.T618T > C	AC-stem	41	aminoacylation	MM	Ling et al 2007
	m.G622G > A	Variable region	45	aminoacylation	EXIT & DEAF	Ling et al 2007
mt-tRNA^Leu(UUR)^ mt-tRNA^Ile^	m.A3243A > G	D-loop	14	aminoacylation 3′ and 5′ end processing wobble base modification	MELAS / LS / DMDF / MIDD / SNHL / CPEO / MM / FSGS / cardiac + multi-organ dysfunction	Rossmanith and Karwan 1998; Yasukawa et al 2000a; Koga et al 2003; Sohm et al 2003; Kirino et al 2004; Levinger et al 2004; Yasukawa et al 2005; Sasarman et al 2008
	m.A3243A > T	D-loop	14	aminoacylation	MM / MELAS / SNHL / CPEO	Sohm et al 2003
	m.C3256C > T	D-stem	25	5′ end processing	MELAS	Rossmanith and Karwan 1998
	m.A3260A > G	AC-stem	29	5′ end processing	MELAS	Rossmanith and Karwan 1998
	m.T3271T > C	AC-stem	40	3′ and 5′ end processing wobble base modification	MELAS / DM	Rossmanith and Karwan 1998; Kirino et al 2004
	m.C3287C > A	T-loop	56	3′ end processing	Encephalomyopathy	Levinger and Serjanov 2012
	m.A3288A > G	T-loop	57	3′ end processing	MM	Levinger and Serjanov 2012
	m.T3291T > C	T-loop	60	3′ end processing	MELAS / MM / DEAF + cognitive impairment	Levinger and Serjanov 2012
	m.A3302A > G	Acc-stem	71	3′ and 5′ end processing	MM	Bindoff et al 1993; Rossmanith and Karwan 1998; Levinger et al 2004
	m.C3303C > T	Acc-stem	72	3′ and 5′ end processing CCA addition	MMC	Koga et al 2003; Levinger et al 2004
	m.A4269A > G	Acc-stem	7	3′ end processing	FICP	Levinger et al 2003
	m.T4274T > C	D-stem	12	aminoacylation 3′ end processing	CPEO/motor neuron disease	Kelley et al 2000; Levinger et al 2003
	m.T4285T > C	AC-stem	27	3′ end processing aminoacylation	CPEO	Kelley et al 2000; Levinger et al 2003
	m.A4295A > G	AC-loop	37	3′ end processing	MHCM / maternally inherited hypertension	Levinger et al 2003
	m.G4298G > A	AC-stem	40	aminoacylation	CPEO/MS	Kelley et al 2000
	m.G4308G > A	T-stem	50	3′ end processing	CPEO	(Schaller et al 2011
	m.G4309G > A	T-stem	51	3′ end processing	CPEO	Levinger et al 2003
	m.A4317A > G	*T*-*loop*	59	3′ end processing CCA-addition aminoacylation	FICP / poss. hypertension factor	Degoul et al 1998; Kelley et al 2001; Levinger et al 2003; Tomari et al 2003; Levinger and Serjanov 2012
	m.C4320 C > T	T-stem	62	3′ end processing	Mitochondrial encephalocardiomyopathy	Levinger et al 2003
mt-tRNA^Met^ and tRNA^Gln^	m.A4401A > G	junction site of tRNAMet and tRNAGln		3′ and 5′ end processing	LVH hypertension + ventricular hypertrophy	Zhu et al 2009
mt-tRNA^Asn^	m.C5703C > T	AC-stem	27	aminoacylation	CPEO / MM	Hao and Moraes 1997
mt-tRNA^Tyr^	m.A5874A > G	D-loop	22	aminoacylation	EXIT	Bonnefond et al 2008
	m.G5877G > A	D-loop	17	aminoacylation	CPEO	Bonnefond et al 2008
mt-tRNA^Ser(UCN)^	m.A7443A > G	pre-tRNA 3′ end		3′ end processing	DEAF	Yan et al 2006a
	m.G7444G > A	pre-tRNA 3′ end		3′ end processing	LHON / SNHL / DEAF	Yan et al 2006b
	m.A7445A > C	pre-tRNA 3′ end		3′ end processing	DEAF	Levinger et al 2001; Yan et al 2006a
	m.A7445A > G	pre-tRNA 3′ end		3′ end processing	SNHL	Yan et al 2006b
	m.7472insC	Variable region	46	aminoacylation 3′ and 5′ end processing	MM / DMDF modulator	Toompuu et al 2002; Toompuu et al 2004
	m.A7480A > G	AC-loop	38	post-transcriptional modification	MM	Yarham et al 2014
	m.C7497C > T	D-stem	22	post-transcriptional modification	MM / EXIT	Mollers et al 2005
	m.A7510A > G	Acc-stem	5	3′ end processing	SNHL	Yan et al 2006a
	m.A7511A > G	Acc-stem	4	3′ end processing	SNHL	Yan et al 2006b
	m.A7512A > G	Acc-stem	3	post-transcriptional modification 3′ end processing	PEM / MERME	Mollers et al 2005; Yan et al 2006a
mt-tRNA^Lys^	m.G8313G > A	D-stem	24	aminoacylation	MNGIE / progressive mito cytopathy	Sissler et al 2004
	m.G8328G > A	AC-stem	39	aminoacylation	Mito encephalopathy / EXIT with MM and ptosis	Sissler et al 2004
	m.A8344 A > G	T-stem	55	wobble base modification	MERRF / LD / depressive mood disorder / leukoencephalopathy / HCM	Yasukawa et al 2000a; Yasukawa et al 2001; Yasukawa et al 2005
	m.T8362 T > G	Acc-stem	71	aminoacylation	MM	Sissler et al 2004
	m.G8363G > A	Acc-stem	72	aminoacylation	MICM + DEAF / MERRF / Autism / LS / Ataxia + Lipomas	Sissler et al 2004; Bornstein et al 2005
mt-tRNA^Gly^	m.A10044A > G	T-loop	59	CCA-addition 3′ end processing	SIDS	Tomari et al 2003; Levinger and Serjanov 2012
mt-tRNA^His^	m.G12192G > A	T-loop	59	3′ end processing	MICM	Levinger and Serjanov 2012
	m.T12201T > C	Acc-stem	68	aminoacylation	DEAF	Gong et al 2014
mt-tRNA^Pro^	m.G15990G > A	AC-loop	36	post-transcriptional modification	MM	Brule et al 1998

List of all known (to our knowledge) disease-causing mtDNA mutations that affect mt-tRNA processing with their possible molecular effect and associated phenotypes. Abbreviations: *CPEO* Chronic progressive external ophthalmoplegia; *DEAF* Maternally inherited deafness; *DM* Diabetes mellitus; *DMDF* Diabetes mellitus & deafness; *EXIT* Exercise intolerance; *FICP* Fatal infantile cardiomyopathy plus, a MELAS-associated cardiomyopathy; *FSGS* Focal segmental glomerulosclerosis; *HCM* hypertrophic cardiomyopathy; *LD*/*LS* Leigh disease/syndrome; *LVH* Left ventricular hypertrophy; *MELAS* Mitochondrial encephalomyopathy, lactic acidosis and stroke-like episodes; MERME MERRF/MELAS overlap disease; *MERRF* Myoclonic epilepsy and ragged red muscle fibers; *MICM* Maternally inherited cardiomyopathy; *MIDD* Maternally inherited diabetes and deafness; *MHCM* Maternally inherited hypertrophic cardiomyopathy; *MM* Mitochondrial myopathy; *MMC* Maternal myopathy and cardiomyopathy; *MNGIE* Mitochondrial neurogastrointestinal encephalopathy disease; *MS* Multiple sclerosis; *PEM* Progressive encephalopathy; *SIDS* Sudden infant death syndrome; *SNHL* Sensorineural hearing loss

#### Primary mutations causing mt-tRNA maturation defects

While mt-tRNA genes only count for approximately 5 % of the total mtDNA sequence, they have been linked with the majority of mitochondrial DNA diseases (Anderson et al 1981; Yarham et al 2010). Different base substitutions in the same mt-tRNA or even the same point mutation can lead to different clinical features. The molecular mechanisms are often poorly understood. Some mutations disrupt transcription factor binding, while others lead to codon recognition problems. The same base substitution can have an effect on more than one maturation step. This has been shown for the MERRF (myoclonic epilepsy with ragged red fibers) m.8344A > G and MELAS m.3243A > G mutation amongst others. We will discuss the molecular mechanism by which these two mutations contribute to pathogenesis only briefly; more details can be found in a review by Yarham et al (2010).

##### Primary mutations interfering with mt-tRNA end processing

Following polycistronic transcription, endonucleolytic 5′- and 3′-end cleavage of precursor mt-tRNA molecules and subsequent 3′-CCA addition are essential to produce mature mt-tRNAs. Several point mutations interfere with one or more of these steps (Table 2, Fig. 6, green, yellow and violet).

Only a few studies have analysed the effect of mt-tRNA mutations on 5′ end leader sequence processing. Ten mutations in the mt-tRNA^Leu(UUR)^ gene were analysed. Four of them, all associated with MELAS, showed a great decrease in RNase P processing, especially m.3256C > T (Moraes et al 1993). This leads to a precursor RNA consisting of 16S mt-rRNA linked to mt-tRNA^Leu(UUR)^ and ND1 as explained above (Rossmanith and Karwan 1998). It also has been shown that m.7472insC impairs 5′ end processing of mt-tRNA^Ser(UCN)^. Interestingly, this insertion also causes 3′ end processing impairment and it was shown that misprocessing at the 3′ end promotes misprocessing at the 5′ end.

Alterations near the mt-tRNA 3′ end could have a deleterious effect on 3′ end endonucleolytic processing. This is indeed observed for tRNA^Leu(UUR)^ mutations m.3303C > T and m.3302A > G as both show a strong interference with 3′ end cleavage (Levinger et al 2004). Interestingly, also non-coding mutations in the pre-tRNA 3′ end of mt-tRNA^Ser(UCN)^ strongly affect the efficiency of 3′ end processing. Four base substitutions were investigated and displayed different effects. m.7443A > G and m.7445A > C gave a strong decrease in ELAC2 efficiency, while another mutation of the same nucleotide (m.7445A > G) has a lower decrease, although cleavage predominantly shifts one nucleotide towards the 3′ end from the normal cleavage site. m.7444G > A shows an increased efficiency, but this cleavage has also shifted by one nucleotide in the 3′ direction of the normal cleavage site (Yan et al 2006a).

Pathogenic substitutions in the mt-tRNA T-arm may also cause impaired 3′ end cleavage, as the T-arm plays an important role in ELAC2 recognition (Hopkinson and Levinger 2008). All mt-tRNAs with a pathogenic mutation in the T-arm studied by Levinger and Serjanov (2012), showed a reduced ELAC2 processing efficiency. The strongest decrease was observed in the mt-tRNA^Ile^ m.4317A > G mutation. This effect on 3′ end cleavage is presumably due to a structural change in the T-arm that impairs precursor binding to ELAC2 (Levinger and Serjanov 2012). Another study analysed the effect of pathogenic mutations in mt-tRNA^Ile^ on 3′ end cleavage. Ten base substitutions were investigated and they all showed a reduced ELAC2 processing, confirming the importance of the T-arm in 3′ end cleavage. However, the mt-tRNA^Ile^ m.4269A > G mutation, located in the acceptor stem, caused the greatest decrease in 3′ processing efficiency. Other substitutions in the acceptor stem such as m.7512A > G, m.7511A > G and m.7510A > G in mt-tRNA^Ser(UCN)^ also produce a mild decrease in ELAC2 processing (Yan et al 2006b).

CCA addition on the 3′ end of mt-tRNAs is another essential step for mt-tRNA maturation. Only few substitutions are known to impair CCA addition by TRNT1, such as m.4317A > G in mt-tRNA^Ile^ (Tomari et al 2003). However, as mentioned above, this mutation also severely impairs 3′ end cleavage (Levinger and Serjanov 2012). It is speculated that the effect on CCA addition is the result of inappropriate positioning of the tRNA substrate on the enzyme, possibly owing to the structural change in the T-arm. Another mutation that affects CCA addition, m.3303C > T in mt-tRNA^Leu(UUR)^, is located in the acceptor stem and presumably decreases the affinity of the enzyme (Levinger et al 2004).

##### Primary mutations interfering with mt-tRNA aminoacylation

Only mature and correctly folded mt-tRNAs can be aminoacylated. Therefore, many mt-tRNA mutations impair aminoacylation efficiency or lead to mischarging (Fig. 6, red). Mutations that frequently affect mt-tRNA aminoacylation are located in the acceptor stem, such as mt-tRNA^Lys^ m.8362 T > G and m.8363G > A (Sissler et al 2004). D-loop mutations that lead to reduced aminoacylation have also been reported. For example, mt-tRNA^Tyr^ m.5874A > G and m.5877G > A (Bonnefond et al 2008) impair tyrosylation by perturbing the tertiary folding and destabilising the D-loop. Interestingly, two different substitutions of the same nucleotide (position 14) of mt-tRNA^Leu(UUR)^, m.3243A > G and m.3243A > T lead to markedly different losses in leucylation efficiency (10-fold and 300-fold, respectively), possibly due to their function in recognition by its cognate tRNA-aminoacyl synthetase (Asahara et al 1998; Sohm et al 2003). The m.3243A > G MELAS mutation also leads to mischarging and consequently to amino acid misincorporation during mitochondrial translation (Sasarman et al 2008). Mischarging is also suggested to contribute to pathogenesis of another MELAS mutation (m.3271 T > C) (Hayashi et al 1993; Sasarman et al 2008).

As discussed above, mutations in the T-arm are generally more prone to impair 3′ end cleavage, while mutations in the D-loop tend to affect aminoacylation. It is speculated that there might be a reciprocal relationship, at least for tRNA^Leu(UUR)^. Mutations that strongly reduce 3′ end processing seem to have little or no effect on aminoacylation after cleavage, while those with reduced aminoacylation capacity affect 3′ processing the least (Levinger et al 2003). Despite this, it is difficult to predict the pathological mechanism based on the position of the substituted nucleotide. For example, two mutations at nucleotide position 72 of mt-tRNA^Leu(UUR)^ (m.3303C > T) and mt-tRNA^Lys^ (m.8363G > A) have opposite effects (Tuppen et al 2012), with the different mutations either causing impaired 3′ end processing and CCA addition, but with unaffected aminoacylation (Levinger et al 2003; Sohm et al 2003) or a diminished aminoacylation capacity (Bornstein et al 2005), respectively.

Although many mutations have been reported to affect aminoacylation, it is usually unknown whether this is the primary effect of the mutation or the consequence of a structural change in the mt-tRNA caused by the nucleotide substitution. This was shown for mutations in the mt-tRNA^Phe^ gene. All studied pathogenic mutations had an effect on aminoacylation, but only in one of them, m.611G > A, an atypical MERRF mutation (Mancuso et al 2004), this seems to be the primary effect (Ling et al 2007).

##### Primary mutations interfering with mt-tRNA modifications

An important step in the post-transcriptional maturation of mt-tRNA is modification of specific nucleotides (see above, Fig. 6, blue). The following mutations have been associated with a loss of taurinomethyl modification at the wobble base (position 34): two in mt-tRNA^Leu(UUR)^, m.3243A > G and m.3271 T > C (Yasukawa et al 2000a) and one in mt-tRNA^Lys^, m.8344A > G (Yasukawa et al 2000b). Wild-type mt-tRNA^Leu(UUR)^ recognises UUG and UUA, while mt-tRNA^Lys^ translates UUU and UUC. Interestingly, the two mt-tRNA^Leu(UUR)^ mutations were shown to have a severe reduced UUG translation, while UUA recognition was apparently unaffected (Kirino et al 2004), whereas mt-tRNA^Lys^ m.8344A > G leads to a complete loss of codon-anticodon pairing for both UUU and UUC (Yasukawa et al 2001). There are two reported mutations at mt-tRNA position 36, m.15990G > A in mt-tRNA^Pro^ and m.7480A > G in mt-tRNA^Ser(UCN)^, both linked with mitochondrial myopathy, that directly affect modification of the neighbouring nucleotide (position 37) (Brule et al 1998; Yarham et al 2014). Enzymes involved in mt-tRNA maturation, such as MTO1, GTPBP3, MTU1 or TRIT1, must have a broad specificity, so their function might be structure-dependent rather than sequence-dependent. In a wider context, this could explain why several mutations have an effect on multiple levels, since several pathogenic base substitutions introduce structural changes.

## Concluding note

Recent research has revealed the important role of mtRNA post-transcriptional maturation in human mitochondrial disease, with mutations in several nDNA- and mtDNA-encoded genes being identified as the genetic cause for these disorders (Tables 1 and 2). Nonetheless, the mechanistic details for the genesis of pathologies associated with defects in mtRNA metabolism remain poorly understood. It is now time to strengthen our efforts to elucidate the molecular machinery and mechanisms responsible for regulating post-transcriptional stages of mtRNA biology. Special attention should be given to functionally assign novel genes involved in mtRNA metabolism. This point is particularly important for a further development of mitochondrial medicine as, despite recent advances in next-generation sequencing technologies, defining the genetic cause of the many unsolved cases of mitochondrial disease remains a major challenge. This research will help to understand the remarkable heterogeneity of human pathologies associated with defects of mtRNA metabolism (Table 1) and to stimulate further studies aimed at the development of effective treatments for these otherwise untreatable diseases.
